# SPACEE Protocol: “Spiritual Care Competence” in PAlliative Care Education and PracticE: Mixed-Methods Research in the Development of Iberian Guidelines

**DOI:** 10.3390/ijerph20043505

**Published:** 2023-02-16

**Authors:** Carlos Laranjeira, Enric Benito, Maria Anjos Dixe, Monica Dones, Marcela Specos, Ana Querido

**Affiliations:** 1 School of Health Sciences, Polytechnic of Leiria, Campus 2, Morro do Lena, Alto do Vieiro, Apartado 4137, 2411-901 Leiria, Portugal; 2 Centre for Innovative Care and Health Technology (ciTechCare), Rua de Santo André—66–68, Campus 5, Polytechnic of Leiria, 2410-541 Leiria, Portugal; 3 Comprehensive Health Research Centre (CHRC), University of Évora, 7000-801 Évora, Portugal; 4 Faculty of Health Sciences, University of Francisco de Vitória, Carretera Pozuelo a, Av de Majadahonda, 28223 Madrid, Spain; 5 Palliative Care Hospital Support Team, The Ramón y Cajal University Hospital of Madrid, M-607, 9, 100, 28034 Madrid, Spain; 6 Departamento de Enfermería, Facultad de Medicina, Autonomous University of Madrid, Calle del Arzobispo Morcillo, n° 4, 28029 Madrid, Spain; 7 Institute Pallium Latin-American, Bonpland 2287, Buenos Aires 1425, Argentina; 8 Center for Health Technology and Services Research (CINTESIS), NursID, University of Porto, 4200-450 Porto, Portugal

**Keywords:** end-of-life, spiritual care competence, mixed-methods approach, healthcare professionals, educators, Portugal, Spain

## Abstract

Spiritual care requires understanding the spiritual experiences of patients and recognizing their resources and needs. Therefore, educators and practitioners should develop their knowledge and understanding in this regard. Spiritual care helps people overcome their anxieties, worries, and suffering; reduces stress; promotes healing; and encourages patients to find inner peace. To provide comprehensive and appropriate care while upholding human/ethical virtues, the spiritual dimension must be a priority. We aim to develop spiritual care competence guidelines for Palliative Care (PC) education and practice in Portugal and Spain. The study detailed in this protocol paper will include three phases. In phase I, the phenomenon will be characterized and divided into two tasks: (1) a concept analysis of “spiritual care competence”; and (2) a systematic review of interventions or strategies used to integrate spiritual care in PC education and practice. Phase II will entail a sequential explanatory approach (online survey and qualitative interviews) to deepen understanding of the perceptions and experiences of educators, practitioners, and patients/family carers regarding spiritual care in PC education and practice and generate ideas for the next steps. Phase III will comprise a multi-phased, consensus-based approach to identify priority areas of need as decided by a group of experts. Results will be used to produce guidelines for integrating spirituality and spiritual care competence within PC education and practice and synthesized in a white book for PC professionals. The value of this improved examination of spiritual care competence will ultimately depend on whether it can inform the development and implementation of tailored educational and PC services. The project will promote the ‘spiritual care’ imperative, helping practitioners and patients/family carers in their preparedness for End-of-Life care, as well as improving curricular practices in this domain.

## 1. Introduction

Suffering is experienced by people, not only by bodies, and it originates from problems that jeopardize the integrity of the person as a complex biopsychosocial and spiritual being [1,2]. Suffering is highly prevalent in the clinic, especially in life-threatening situations such as palliative care [3]. The current care model is often based on the paradigm of scientific materialism, which given its epistemological limitations, is unable to understand or address this reality [4]. In other words, clinicians do not have a model, framework, or paradigm to fully understand and attend to suffering. Physicians’ failure to comprehend the holistic nature of suffering can result in medical interventions that, while technically appropriate, not only fail to relieve suffering but can cause it [5].

The lack of a comprehensive academic model and the prevalence of the scientific/materialistic paradigm, therefore, produces more suffering not only for patients and their families but also for professionals, which manifests as emotional distress and other mental health problems [6,7]. Given the need to understand and attend to this human experience, especially among health professionals, attempts have been made for some years to expand the limited materialistic framework and integrate spirituality into clinical practice [8].

Spirituality, according to the European Association for Palliative Care, “is the dynamic dimension of human life that relates to how people experience, express, and/or seek meaning, purpose, and transcendence, as well as how they connect to the moment, to self, to others, to nature, to the significant, and/or to the sacred” [9] p. 2. This term encompasses a broad understanding of spirituality, implying that a person can rely on a variety of sources of spiritual connection [10]. Spiritual care is the integration of spirituality into the therapeutic situation with a focus on healing the whole person [11,12,13].

Nowadays, spiritual care is seen as a crucial piece of total care provided to patients in order to improve their overall health, quality of life, and well-being [12,14,15]. The integration of spiritual care standards provides practitioners with a greater understanding of their patients, enhancing their potential to inspire hope and relieve suffering [16,17]. Despite increased interest in spiritual care within health sciences education and practice [18], it remains the least developed and most neglected dimension of care, particularly Palliative Care (PC) [13].

Spiritual care competency is a vital skill for all health care providers, including in nursing, medicine, and psychology. Notwithstanding, there is little to no evidence of spiritual care competency in PC training and practice. Several obstacles to incorporating spirituality into healthcare have been identified [19,20,21], including inadequate staff training [22,23], a belief that spiritual care is ‘not my professional role’ [24], “peer pressure, a perceived lack of time due to prioritization of other medical or physical concerns, confusion between spirituality and religion, and staff members’ lack of comfort with, or awareness of, their own personal spirituality” [10] p. 262.

Although many articles and some international consensus documents have been published, there is no complete agreement on how to address spiritual care competence and the obstacles to its implementation. The international EPICC (Enhancing Nurses’ and Midwives’ Competence in Providing Spiritual Care through Innovative Education and Compassionate Care) project network revealed substantial variance in how spirituality is handled in nurse and midwifery education programs throughout Europe [25,26]. The initiative resulted in standards of excellence for spiritual care education and led to the development of learning materials and evaluation of competencies (knowledge, skills, and attitudes) to provide spiritual care. Considering the cultural differences and spiritual beliefs across different countries, spiritual care is not transferable wholesale [27]. This means that general principles may be transferable, but there is a need for local modifications. In addition, the evidence highlights the need for a more unified and standardized method for gaining spiritual care competencies in multidisciplinary educational and practice settings [27].

In general, PC practitioners lack the knowledge, skills, or time to provide adequate spiritual care [28,29]. Despite this, they are mostly aware that providing spiritual care is part of their role [8,30]. While a considerable amount of literature has been published on the role of spiritual care in PC practice, in particular about identifying spiritual needs and offering appropriate care [31,32,33,34], there are gaps in PC theory and practice on the importance of spiritual care competence [13]. To circumvent or mitigate these constraints, PC education and practice should be founded on an existential-humanistic approach that promotes a holistic view of a person and human flourishing [35]. This framework would stimulate the practitioner’s awareness of how to foster individual experiences of existential or spiritual strength and improve the opportunities for personal growth, well-being, and satisfaction [35].

Scarce research has investigated this phenomenon and developed accurate criteria for integrating spiritual care in PC practice and education [36], thereby bridging the gap between theory and practice. To address this gap, the primary purpose of this project is to develop spiritual care competence recommendations for PC education and practice in Portugal and Spain. Some secondary objectives were also defined: (a) a concept analysis of the role of “spiritual care competence” in PC education and practice; (b) a systematic review of the interventions or strategies used to integrate spiritual care in PC education and practice; (c) a study of the perceptions and experiences of educators, practitioners, and patients/family carers regarding spiritual care competence in PC education and practice; and (d) validated guidelines for integrating spiritual care competence in PC education and practice.

We hope that our guidelines contribute to delivering culturally and spiritually sensitive quality PC in Iberia that emphasizes the relevance of person-centered care and enhances spiritual care competence as a central part of professional development.

## 2. Materials and Methods

### 2.1. Study Overview

To achieve our goals, the SPACEE study will adopt a multi-phase mixed methods triangulation design, which integrates parallel and/or sequential qualitative and quantitative data-gathering methodologies throughout three or more stages, usually throughout a multi-year project [37]. In iterative multi-phase designs, each new step of data collecting informs the conceptualization and application of the next phase. Data triangulation is a cornerstone of mixed methods research, offering a comprehensive view [38] that can improve the credibility of research findings. The study’s stages and data-gathering procedures are intended to accomplish robust data triangulation. We will triangulate data by data source (documents and reports, different target samples) and method (literature review, survey, interview).

The project will be led by a multidisciplinary panel composed of 6 experts from the fields of Medicine (E.B.), Nursing (C.L.; A.Q.; M.A.D.; M.D.), and Pedagogy (M.S.), each with at least 10 years of experience in education and research in Palliative Care.

**Figure 1:**
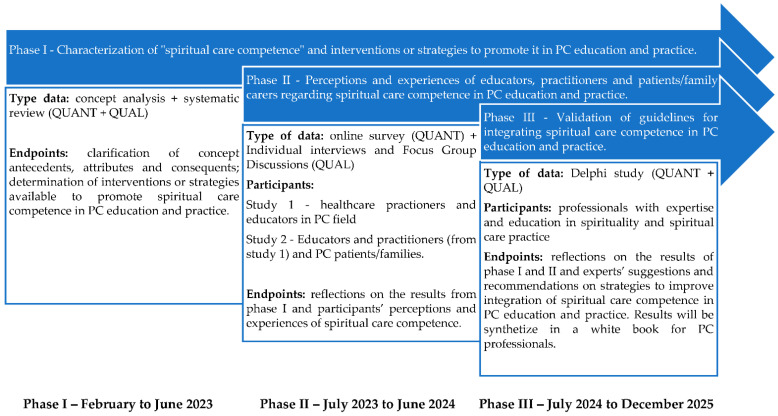
Study overview.

Figure 1 depicts data triangulation during the different study phases, as well as how these phases are linked. The study will begin in February 2023. The estimated completion date for the study is December 2025.

This study protocol was prospectively registered in Open Science Framework (OSF) on 14 September 2022 (osf.io/4vxwu).

#### 2.1.1. Phase I

##### Purpose

Spiritual care competency is a core ability for all caregiving professionals to deliver holistic care. However, the antecedents, attributes, and outcomes of spiritual care competence in PC training and practice are unclear. To address this gap, we will conduct a concept analysis of “spiritual care competence” (Task 1). Concept analysis is an effective approach for finding gaps in available knowledge and developing inferences about the overall condition of research activities.

Since “competency-based spiritual care education, practical training and maintaining the link between spiritual care education and clinical practice” [39] p. 1 are required, we will also develop a systematic review of interventions or strategies used to integrate spiritual care competence in PC education and practice (Task 2).

This strategy will enable our team to obtain relevant, readily available material in order to define state of the art in the phenomena under investigation. These data will also be used to create key informant web-based surveys and semi-structured interview guides for use in subsequent study stages.

##### Procedures

Task 1: Concept analysis on “Spiritual Care Competence” in PC.

A Pragmatic Utility concept analysis based on relevant literature will be performed [40]. A rigorous process of asking and answering analytical questions will establish a definition of the concept and determine its antecedents, attributes, boundaries, and outcomes. Electronic searches will be conducted using the databases CINAHL, Pubmed, Scopus, Cochrane, and Science Direct. The search terms (‘spiritual*’ OR ‘religio*’ OR ‘existenti*’) will be combined with (‘care’ OR ‘competenc*’) AND (‘palliative care’ OR ‘terminal care’ OR ‘terminally ill’) with no time restrictions on published articles. To ensure relevance for PC educators and practitioners, only scholarly, peer-reviewed studies with full text in English, Spanish, and Portuguese will be included. The PRISMA Protocol checklist [41] will be used.

Task 2: Systematic review.

We will search six databases: Web of Science, Medline/PubMed, Cochrane, PsycINFO, Scopus, and CINAHL. Systematic searches will be conducted by combining the following terms: spiritual* *AND* strateg* and intervention* and spiritual care competence *AND* palliative care *AND* education and practice. Eligible articles will be included if they (1) studied participants who are adults (≥18 years old); (2) include empirical research with qualitative, quantitative, or mixed-method study design; (3) strategies or interventions related to teaching, learning or exercising spiritual care competence; (4) palliative care related; (5) scholarly papers, peer-reviewed; and (6) full-text articles in English, Spanish, and Portuguese languages. The searches will not be time-constrained.

This review will be conducted according to the PRISMA 2020 statement [41]. References found in the database search will be added to Rayyan Software [42], and duplicates and cross-references will be removed. The titles, abstracts, and keywords of the selected papers will be screened by two independent reviewers using the above-mentioned eligibility criteria. Eligible full-text manuscripts and articles with unclear inclusion based on the abstracts will be evaluated separately by the same reviewers to confirm inclusion or exclusion. Disagreements will be resolved during a consensus meeting. A third reviewer will be consulted if a consensus cannot be reached. The inclusion or removal of studies will be reported independently by two researchers using Excel spreadsheet software.

Methodological quality will be assessed by two independent reviewers, using either the Critical Appraisal Skills Program [43] checklists or Mixed Methods Appraisal Tools [44]. A narrative approach will be used to undertake a qualitative synthesis of the body of research. A meta-analysis will be conducted if necessary, depending on the degree of variability across included studies. The protocol for the systematic review will be submitted to the PROSPERO registry for assessment and approval and will be described in the systematic study’s final report.

#### 2.1.2. Phase II

##### Purpose

The dearth of practice and formal education about spiritual care competence poses challenges for preparing educators with the required teaching skills and practitioners who need to address patients’ spiritual matters. To fill the current gap in spiritual care education and practice, we will conduct a mixed-methods sequential explanatory design [45,46], composed of two parts: first, an online survey with educators and practitioners in PC (Task 3); second, an in-depth qualitative study with patients and families and a Focus Group Discussion with educators and practitioners in PC (Task 4). Quantitative and qualitative data will be collected sequentially [35]. Using merging procedures, participant narratives will provide a more thorough understanding of the statistical findings [47].

##### Procedures

Task 3a: Methodological Pilot Study

A pilot study will be conducted to assess the validity and reliability of the Spirituality Care Competency Scale (SCCS). The SCCS consists of six subdomains: “assessment and implementation of spiritual care, professionalization and improvement of the quality of spiritual care, personal support and patient counseling, referral to professionals, attitude toward patients’ spirituality, and communication” [48] p. 2. There are 27 items that are answered on a 5-point Likert scale. The overall score ranged between 27 and 135. A score < 64 indicates a low level of competence, a score of 64–98, an average level, and a score ≥ 99, a high level [49].

The SCCS Tool (originally in English) will be translated into Portuguese and Spanish using a forward-backward translation method based on the International Test Commission [50] stepwise procedure (see Table 1).

To establish the initial psychometric properties of the questionnaire, at least 300 participants with experience in PC (nurses, psychologists, social workers, physicians, chaplains, and spiritual assistants) will be recruited through a convenience sampling method across two hospitals and two community palliative care services from both countries. We assume that the optimal sample would be ten respondents per scale item. Tabachnick and Fidell [51] recommend 300 cases as a good rule of thumb for factor analysis. Participants from the pilot study will not be entered into the subsequent survey (task 3b).

Reliability, face validity, content validity, and construct validity (factorial analysis) of the instrument will be applied in the study. Data will be analyzed using SPSS-28 [52].

Task 3b: A multicenter, quantitative descriptive cross-sectional survey.

The STROBE checklist [53] will be followed. Convenience sampling will be used to recruit educators who teach in PC advanced programs at higher education institutions in Portugal and Spain (at least 50 in each country). Convenience sampling will also be used to recruit registered practitioners from the Portuguese and Spanish PC Units (Hospitals and Community settings). To meet the statistical requirements, the investigators will recruit a minimum of 500 participants. Data will be collected via an online self-report questionnaire (Google form) through the mailing lists and intranet of the Portuguese Association of Palliative Care [APCP] and the Spanish Society of Palliative Care [SECPAL].

Validated instruments will collect data for those variables that may influence competence in spiritual care: self-knowledge (spiritual well-being), beliefs (coping with death), and individual perceptions about spirituality. The e-survey consists of 5 sections: (a) demographics (sex, age, education level, marital status, job seniority, religious affiliation, place and nature of employment, and training in spiritual care); (b) Spiritual Well-being Questionnaire [54] [to measure personal, communal, environmental, and transcendental spiritual well-being]; (c) Coping with Death Scale [55] [to access skills for facing death, as well as beliefs and attitudes about these capacities]; (d) Spirituality and Spiritual Care Rating Scale [56] [to identify the perceptions towards spirituality and spiritual care]; and (e) Spirituality Care Competency Scale (SCCS) [57] that was reported to be reliable in previous studies [58], but lacks Portuguese and Spanish validation. All instruments have Portuguese and Spanish versions except the SCCS, which will be adapted and validated for this project (task 3a).

Task 4: A multisite, descriptive, and contextual qualitative study.

Educators and practitioners from the cross-sectional survey (task 3) who consent will be invited to participate in a qualitative interview (via email). Purposive sampling will be used to recruit educators and practitioners in PC (n = 50) (should achieve data saturation), with 25 participants from each country. No restrictions will be placed on their gender, age, or seniority.

Purposive sampling will also be used to recruit PC patients/family carers (n = 30) (should achieve data saturation) from four PC Units (Hospital and Community) (two units from each country). Permission to conduct this study will be previously requested from the facility manager of these sites. Data will be collected via individual interviews with patients and focus group discussions with educators and practitioners. The interviews will shed light on the previously gathered quantitative data. The interview guide will address participants’ (patients/family caregivers, educators, and practitioners) perspectives on spiritual care needs and preferences, spiritual care competency within PC practice, and facilitators/barriers to spiritual care attendance. Trained research staff will perform all interviews. The interviews will be audio-recorded and transcribed verbatim before being imported into WebQDA software [59].

Qualitative data will be analyzed using thematic analysis [60]. The first phase, familiarization, entails in-depth understanding and involvement with the data. The second phase, coding, involves detecting and labeling significant data features. The third phase implies looking for themes and concentrating the codes on an overarching topic. Next, the themes are reviewed—in two parts: (i) in relation to the collected, coded data for each topic and (ii) in relation to the data set. Then, the themes are named and defined by identifying the core of each theme. Lastly, there is the final analysis and writing up of results. Long-term participation in data collection until data saturation will assure legitimacy. All participating researchers will be subjected to member verification and peer debriefing. The study will follow the COREQ checklist [61].

#### 2.1.3. Phase III

##### Purpose

We will synthesize findings from phase I and phase II to produce draft guidelines (Task 5) for integrating spiritual care competence in PC education and practice in Portugal and Spain. Then, we will document and synthesize evidence-based recommendations for spiritual care competency in PC education and practice through a White Book (Task 6).

##### Procedures

Task 5: A multi-phase modified Delphi method

A Delphi study [62] with three rounds will be undertaken. Based on the findings of previous studies, the research team will provide the first proposal of spiritual care competency criteria for PC practice and education. This will provide specialists with the knowledge they need to create ideas and topics regarding the phenomena. The proposal will be emailed to experts who will assess the summarized items and rank them in terms of priority. A consensus among expert ranks will be sought.

Participating experts will be chosen based on the following criteria: (a) they must be Spanish or Portuguese; (b) they have education and expertise in spiritual care practice; (c) a master’s degree and a senior position in education or palliative clinical practice; and (d) research and publications on spiritual care. A purposive sample of 40 experts will be recruited through the APCP and SECPAL. Experts will be invited to provide informed consent to participate in this study.

In a face-to-face workshop, experts will discuss the explanations, clarifications, and justifications for the items ranked earlier. This information will then be analyzed and used to further modify the proposal and reach the final guidelines.

In the final phase, 10 experts who participated in earlier rounds of the study will form an expert review panel and assess the Delphi findings in a two-step procedure. In Step 1, the panel will evaluate and comment on the created items and raise any major concerns they believe were overlooked. In Step 2, the panel will rank items from 1–10 for inclusion in the final report, eliminating the remaining items. The panel’s completed forms will be returned electronically.

Items will be accepted “if at least 70% of caregiver and professional panels assessed an item as (4) ‘important’ or (5) ‘extremely important.’ Items will be re-rated if 70% of one panel or the overall sample evaluated an item as (4) ‘important’ or (5) ‘extremely important,’ but the other panel did not, indicating disagreement between the panels. Items will be rejected if it did not fulfill the 70% requirement in both panels or the entire sample” [63] p. 231.

Task 6: White Book

Based on results from previous tasks, a White Book will be designed to document and synthesize evidenced-based recommendations for spiritual care competency in PC education and practice in Portugal and Spain. This book will represent a position statement regarding spiritual care competency in PC, intended to be used for advocacy with local governments, educational and healthcare organizations, and leaders on the ground. We will also outline the challenges faced by educators and practitioners and suggest optimal ways for providers, stakeholders, and decision-makers to implement and evaluate spiritual care competency. The White Book will be written in English, Spanish, and Portuguese languages.

### 2.2. Ethical Considerations

The SPACEE study was examined and will be carried out in accordance with the principles of the Helsinki Declaration, and the protocol was approved by the Polytechnic of Leiria’s Research Ethics Committee (approval no 50/2022). Participants will be informed that they might opt out of the study at any time. They will not be financially compensated for their participation. Professional assistance will be accessible if patients or family carers desire, given their physical and psychological vulnerability. All data stored electronically will be kept on a computer (password protected) to keep the research data confidential and limited to the research team. The research data and records will be maintained for 5 years after publication.

## 3. Discussion

The project will contribute to valuing spiritual care in PC while promoting a paradigm shift in medicalized healthcare toward a person-centered approach that recognizes the need to respect and empower patients [64]. This paradigm shift recognizes spirituality as an inner resource for health that improves patient outcomes such as healing, growth, coping, hope, and purpose [65]. Competencies are necessary to guarantee that spiritual care and practice are person-centered, timely, effective, and safe in the environment in which they are provided, with the overarching goal of improving patient welfare [65]. Using a competency-based learning approach assists in providing healthcare workers with the necessary knowledge, abilities, and attitudes to practice with confidence [66]. We also believe that this study (from a multi-professional stance) will provide a foundation for the Iberian context to clearly define the skills necessary for spiritual care, how to teach them, and how to apply and evaluate them in the PC field. Our ultimate aim includes dissemination of our findings in peer-reviewed journals, professional conferences, and through brief reports for participating entities and stakeholders. The participation of the associations APCP and SECPAL will be crucial to this purpose.

Notwithstanding, our study does have potential limitations. Although we will use established techniques (e.g., integrating multiple sources of data and methods) to improve the rigor and generalizability of our analysis, our study is limited by the fact that it will take place in selected PC units and will be focused on purposive samples of educators and practitioners assigned in national associations of PC.

## 4. Conclusions

This study protocol will offer an extensive review of the “state-of-art” regarding spiritual care competence as a priority for healthcare professionals in their PC education and practice. Furthermore, the development of Iberian guidelines for spiritual care competence will result in the development of collaborative deliberation between professionals and patients in Portugal and Spain, resulting in well-informed and preference-based decisions that promote intentional and proactive care of the spirit. Moreover, the promotion of spiritual care competence will result in safer and more patient-centered PC in Iberia, including improvements in health outcomes.

## Figures and Tables

**Table 1:** Forward-backward translation procedure [50].

**Step 1**	Two independent translators (familiar with both languages and the instrument’s aims) create two separate forward translations of the tool
**Step 2**	Reconciliation into single translation by project leader
**Step 3**	Back-translation into source language by two independent translators with proficiency in both languages
**Step 4**	A panel composed of 6 experts (practitioners, educators, researchers, subject area experts, and a tool validation expert) review the process and validate the final version in the target language

## Data Availability

Not applicable.

## References

[1] Binder P.E. (2022). Suffering a Healthy Life—On the Existential Dimension of Health. Front. Psychol..

[2] Hartogh G.D. (2017). Suffering and dying well: On the proper aim of palliative care. Med. Health Care Philos..

[3] Siler S., Borneman T., Ferrell B. (2019). Pain and Suffering. Semin. Oncol. Nurs..

[4] Rocca E., Anjum R.L., Anjum R., Copeland S., Rocca E. (2020). Complexity, Reductionism and the Biomedical Model. Rethinking Causality, Complexity and Evidence for the Unique Patient.

[5] Benito E., Dones M., Barbero J. (2017). El acompañamiento espiritual en cuidados paliativos. Psicooncologia.

[6] Soto-Rubio A., Perez-Marin M., Tomas Miguel J., Barreto Martin P. (2018). Emotional Distress of Patients at End-of-Life and Their Caregivers: Interrelation and Predictors. Front. Psychol..

[7] Søvold L.E., Naslund J.A., Kousoulis A.A., Saxena S., Qoronfleh M.W., Grobler C., Münter L. (2021). Prioritizing the Mental Health and Well-Being of Healthcare Workers: An Urgent Global Public Health Priority. Front. Public Health.

[8] Dones Sánchez M., Bimbaum N.C., Barbero Gutierrez J., Bofill C., Balbuena Mora-Figueroa P., Benito E. (2016). How professionals perceive spiritual care in palliative care teams in Spain?. Med. Paliativa.

[9] Best M., Leget C., Goodhead A., Paal P. (2020). An EAPC white paper on multi-disciplinary education for spiritual care in palliative care. BMC Palliat. Care.

[10] Jones K.F., Paal P., Symons X., Best M.C. (2021). The Content, Teaching Methods and Effectiveness of Spiritual Care Training for Healthcare Professionals: A Mixed-Methods Systematic Review. J. Pain Symptom Manag..

[11] Van Leeuwen R., Schep-Akkerman A. (2015). Nurses’ perceptions of spirituality and spiritual Care in Different Health Care Settings in the Netherlands. Religions.

[12] Cooper K.L., Chang E., Luck L., Dixon K. (2020). How Nurses Understand Spirituality and Spiritual Care: A Critical Synthesis. J. Holist. Nurs..

[13] Gijsberts M.H.E., Liefbroer A.I., Otten R., Olsman E. (2019). Spiritual Care in Palliative Care: A Systematic Review of the Recent European Literature. Med. Sci..

[14] Atarhim M.A., Lee S., Copnell B. (2019). An Exploratory Study of Spirituality and Spiritual Care Among Malaysian Nurses. J. Relig. Health.

[15] García-Navarro E.B., Medina-Ortega A., García Navarro S. (2021). Spirituality in Patients at the End of Life-Is It Necessary? A Qualitative Approach to the Protagonists. Int. J. Environ. Res. Public Health.

[16] Laranjeira C., Querido A., Charepe Z., Dixe M.A. (2020). Hope-based interventions in chronic disease: An integrative review in the light of Nightingale. Rev. Bras. Enferm..

[17] Laranjeira C., Baptista Peixoto Befecadu F., Da Rocha Rodrigues M.G., Larkin P., Pautex S., Dixe M.A., Querido A. (2022). Exercising Hope in Palliative Care Is Celebrating Spirituality: Lessons and Challenges in Times of Pandemic. Front. Psychol..

[18] Yildirim J.G., Ertem M. (2022). Professional quality of life and perceptions of spirituality and spiritual care among nurses: Relationship and affecting factors. Perspect. Psychiatr. Care.

[19] Benito E., Barbero J., Dones M., SECPAL (2014). Espiritualidad en Clínica Una Propuesta de Evaluación y Acompañamiento Espiritual en Cuidados Paliativos.

[20] Best M., Butow P., Olver I. (2016). Doctors discussing religion and spirituality: A systematic literature review. Palliat. Med..

[21] Bar-Sela G., Schultz M.J., Elshamy K., Rassouli M., Ben-Arye E., Doumit M., Gafer N., Albashayreh A., Ghrayeb I., Turker I. (2019). Training for awareness of one’s own spirituality: A key factor in overcoming barriers to the provision of spiritual care to advanced cancer patients by doctors and nurses. Palliat. Support Care.

[22] Jones K.F., Pryor J., Care-Unger C., Simpson G.K. (2020). Rehabilitation health professionals’ perceptions of spirituality and spiritual care: The results of an online survey. Neuro Rehabil..

[23] Kang K.A., Chun J., Kim H.Y., Kim H.Y. (2021). Hospice palliative care nurses’ perceptions of spiritual care and their spiritual care competence: A mixed-methods study. J. Clin. Nurs..

[24] Balboni M., Sullivan A., Enzinger A., Epstein-Peterson Z., Tseng Y., Mitchell C., Niska J., Zollfrank A., VanderWeele T., Balboni T. (2014). Nurse and physician barriers to spiritual care provision at the end of life. J. Pain Symptom Manag..

[25] McSherry W., Ross L., Attard J., van Leeuwen R., Giske T., Kleiven T., Boughey A., The EPICC Network (2020). Preparing undergraduate nurses and midwives for spiritual care: Some developments in European education over the last decade. J. Study Spiritual..

[26] Van Leeuwen R., Attard J., Ross L., Boughey A., Giske T., Kleiven T., McSherry W. (2021). The development of a consensus-based spiritual care education standard for undergraduate nursing and midwifery students: An educational mixed methods study. J. Adv. Nurs..

[27] Rykkje L., Søvik M.B., Ross L., McSherry W., Cone P., Giske T. (2022). Educational interventions and strategies for spiritual care in nursing and healthcare students and staff: A scoping review. J. Clin. Nurs..

[28] Benito E., Oliver A., Galiana L., Barreto P., Pascual A., Gomis C., Barbero J. (2014). Development and validation of a new tool for the assessment and spiritual care of palliative care patients. J. Pain Symptom Manag..

[29] Chahrour W.H., Hvidt N.C., Hvidt E.A., Viftrup D.T. (2021). Learning to care for the spirit of dying patients: The impact of spiritual care training in a hospice-setting. BMC Palliat. Care.

[30] Guedes A., Carvalho M.S., Laranjeira C., Querido A., Charepe Z. (2021). Hope in palliative care nursing: Concept analysis. Int. J. Palliat. Nurs..

[31] Koper I., Pasman H.R.W., Schweitzer B.P.M., Kuin A., Onwuteaka-Philipsen B.D. (2019). Spiritual care at the end of life in the primary care setting: Experiences from spiritual caregivers—A mixed methods study. BMC Palliat. Care.

[32] Fitch M.I., Bartlett R. (2019). Patient Perspectives about Spirituality and Spiritual Care. Asia Pac. J. Oncol. Nurs..

[33] Batstone E., Bailey C., Hallett N. (2020). Spiritual care provision to end-of-life patients: A systematic literature review. J. Clin. Nurs..

[34] Ghorbani M., Mohammadi E., Aghabozorgi R., Ramezani M. (2021). Spiritual care interventions in nursing: An integrative literature review. Support. Care Cancer.

[35] Haufe M., Leget C., Potma M., Teunissen S. (2020). How can existential or spiritual strengths be fostered in palliative care? An interpretative synthesis of recent literature. BMJ Support. Palliat. Care.

[36] Baldacchino D. (2015). Spiritual Care Education of Health Care Professionals. Religions.

[37] Creswell J., Plano Clark V. (2018). Designing and Conducting Mixed Methods Research.

[38] O’Cathain A., Murphy E., Nicholl J. (2010). Three techniques for integrating data in mixed methods studies. BMJ.

[39] Paal P., Roser T., Frick E. (2014). Developments in spiritual care education in German-speaking countries. BMC Med. Educ..

[40] Morse J.M. (2016). Analyzing and Conceptualizing the Theoretical Foundations of Nursing.

[41] Page M.J., McKenzie J.E., Bossuyt P.M., Boutron I., Hoffmann T.C., Mulrow C.D., Shamseer L., Tetzlaff J.M., Akl E.A., Brennan S.E. (2021). The PRISMA 2020 statement: An updated guideline for reporting systematic reviews. BMJ.

[42] Ouzzani M., Hammady H., Fedorowicz Z., Elmagarmid A. (2016). Rayyan-a web and mobile app for systematic reviews. Syst. Rev..

[43] Critical Appraisal Skills Programme (2018). CASP Checklists. https://casp-uk.net/casp-tools-checklists/.

[44] Hong Q.N., Pluye P., Fàbregues S., Bartlett G., Boardman F., Cargo M., Dagenais P., Gagnon M.-P., Griffiths F., Nicolau B. (2018). Mixed Methods Appraisal Tool (MMAT), Version 2018.

[45] Creswell J.W., Plano Clark V.L., Gutmann M.L., Hanson W.E., Tashakkori A., Teddlie C. (2003). Advanced mixed methods research design. Handbook of Mixed Methods in Social and Behavioral Research.

[46] Draucker C.B., Rawl S.M., Vode E., Carter-Harris L. (2020). Integration Through Connecting in Explanatory Sequential Mixed Method Studies. West. J. Nurs. Res..

[47] Fetters M.D., Curry L.A., Creswell J.W. (2013). Achieving integration in mixed methods designs—Principles and practices. Health Serv. Res..

[48] Fang H.F., Susanti H.D., Dlamini L.P., Miao N.F., Chung M.H. (2022). Validity and reliability of the spiritual care competency scale for oncology nurses in Taiwan. BMC Palliat. Care.

[49] Abusafia A.H., Mamat Z., Rasudin N.S., Bakar M., Ismail R. (2021). Spiritual care competence among Malaysian staff nurses. Nurse Media J. Nurs..

[50] International Test Commission (2017). International Test Commission Guidelines for Translating and Adapting Tests.

[51] Tabachnick B., Fidell L. (2013). Using Multivariate Statistics.

[52] IBM Corp (2021). IBM SPSS Statistics for Windows, Version 28.0.

[53] Von Elm E., Altman D.G., Egger M., Pocock S.J., Gøtzsche P.C., Vandenbroucke J.P., STROBE Initiative (2007). The Strengthening the Reporting of Observational Studies in Epidemiology (STROBE) statement: Guidelines for reporting observational studies. Prev. Med..

[54] Gomez R., Fisher J. (2003). Domains of Spiritual Well-Being and Development and Validation of the Spiritual Well-Being Questionnaire. Pers. Individ. Dif..

[55] Bugen L. (1981). Coping: Effects of death education. Omega.

[56] McSherry W., Draper P., Kendrick D. (2002). The construct validity of a rating scale designed to assess spirituality and spiritual care. Int. J. Nurs. Stud..

[57] Van Leeuwen R., Tiesinga L.J., Middel B., Post D., Jochemsen H. (2009). The validity and reliability of an instrument to assess nursing competencies in spiritual care. J. Clin. Nurs..

[58] Hu Y., Leeuwen R.V., Li F. (2019). Psychometric properties of the Chinese version of the spiritual care competency scale in nursing practice: A methodological study. BMJ Open.

[59] Sousa F.N., Costa A.P., Moreira A. (2019). webQDA [Software].

[60] Braun V., Clarke V. (2006). Using thematic analysis in psychology. Qual. Res. Psychol..

[61] Tong A., Sainsbury P., Craig J. (2007). Consolidated criteria for reporting qualitative research (COREQ): A 32-item checklist for interviews and focus groups. Int. J. Qual. Health Care.

[62] McKenna H.P. (1994). The Delphi technique: A worthwhile research approach for nursing?. J. Adv. Nurs..

[63] Knighting K., O’Brien M.R., Roe B., Gandy R., Lloyd-Williams M., Nolan M., Jack B. (2016). Gaining consensus on family carer needs when caring for someone dying at home to develop the Carers’ Alert Thermometer (CAT): A modified Delphi study. J. Adv. Nurs..

[64] Demirsoy N. (2017). Holistic Care Philosophy for Patient-Centered Approaches and Spirituality. Patient-Centered Medicine.

[65] Vincensi B.B. (2019). Interconnections: Spirituality, Spiritual Care, and Patient-Centered Care. Asia Pac. J. Oncol. Nurs..

[66] Kelly E., Cobb M.R., Puchalski C.M., Rumbold B. (2012). Competences in spiritual care education and training. Oxford Textbook of Spirituality in Healthcare.

